# Economic cost of a case of diarrhoea in Uvira, Democratic Republic of the Congo: A cost of illness study

**DOI:** 10.1371/journal.pntd.0011934

**Published:** 2024-10-28

**Authors:** Patrick V. Katana, Espoir Bwenge Malembaka, Patrick Musole Bugeme, Jaime Mufitini Saidi, Oliver Cumming, Karin Gallandat, Ian Ross

**Affiliations:** 1 Department of Disease Control, London School of Hygiene & Tropical Medicine, Keppel St, London, United Kingdom; 2 Department of Epidemiology, Johns Hopkins Bloomberg School of Public Health, Johns Hopkins University, Baltimore, Maryland, United States of America; 3 Center for Tropical Diseases and Global Health, Université Catholique de Bukavu, Bukavu, Democratic Republic of the Congo; 4 Ministère de la Santé Publique, Division Provinciale de la Santé Publique, District Sanitaire d’Uvira, Uvira, South Kivu, Democratic Republic of the Congo; Wayne State University, UNITED STATES OF AMERICA

## Abstract

**Background:**

Diarrhoea is one of the leading causes of disability-adjusted life years (DALYs) among children below five years, though the proportion of the burden occurring amongst those aged over 70 is increasing. The cost of treating and managing diarrhoea can place a burden on individuals, their households, and society in general. The cost can be high but is often undocumented, and many studies focus on children or hospitalised patients only. This study aimed to estimate the economic cost per case of diarrhoea amongst individuals of any age in Uvira, Democratic Republic of the Congo.

**Methods:**

The study was cross-sectional and retrospective, and based on a household survey approximately representative of the city undertaken in September 2021. Data on quantities and prices of resources were collected in the survey, as well as from interviews with staff at the Cholera Treatment Centre in the Uvira general hospital and their records on resource use and patient numbers. Direct and indirect costs were measured from the societal perspective, and generalised linear regression used to identify factors associated with higher costs.

**Results:**

Of 2,820 members of the 528 households surveyed, 175 people (6.2%) were reported to have had diarrhoea in the previous seven days. The majority sought care (91%) of which most (64%) visited a pharmacy. The average economic cost of illness (COI) for an episode of diarrhoea was 33,816 Congolese Francs (CDF) (US$ 17.0) in 2021 prices. The median was CDF 14,000 (US$ 7.0). The average out of pocket COI to patients was CDF 15,579 (US$ 7.8), representing 9% of the estimated average monthly income of households. On average caregivers and patients lost 4 days per episode. A concentration index suggested a lower economic COI among poorer households (p=0.099). A regression analysis identified that being older than 5 years (p=0.001) or being water insecure (p=0.032) were associated with higher COI.

**Conclusion:**

Households in Uvira experience many diarrhoeal episodes per year, and the COI is an important burden for them and society. These costs could be avoided if diarrhoea were prevented through public health interventions to reduce prevalence and care-seeking was better managed to avert this public health burden.

## Author summary

The cost of treating and managing diarrhoea can place a burden on individuals, their households, and society in general. The cost is often undocumented, and many studies focus on children or hospitalised patients only. We estimated the cost borne per case of diarrhoea in Uvira, Democratic Republic of the Congo, by asking people with family members experiencing diarrhoea in the past 7 days about the costs they faced. Out of 2,820 members of the 528 households we interviewed overall, 175 people (6.2%) had diarrhoea in the previous 7 days. Most (64%) visited a pharmacy to seek care. The average economic cost per episode of diarrhoea was 33,816 Congolese Francs (CDF) (US$ 17.0) in 2021 prices, including both money spent out of pocket and indirect costs (e.g. lost caregiver or patient wages). The average out of pocket cost was CDF 15,579 (US$ 7.8). These costs could be avoided if diarrhoea were prevented through public health interventions to reduce prevalence and care-seeking was better managed to avert this public health burden.

## Introduction

Despite concerted effort in the fight against diarrhoea, this cluster of diseases remains a serious public health problem globally [1,2]. Diarrhoea was estimated to be the third leading cause of disability-adjusted life years (DALYs) among children below five years, with an increasing burden amongst those aged 70 and above [3]. Low-income settings in Sub-Saharan Africa and South Asia bear the highest burden [2,4]. Important risk factors for diarrhoea mortality and morbidity include limited access to healthcare, lack of access to clean water, lack of rotavirus vaccine availability, undernutrition, and stunting [5]. Much diarrhoea is preventable by personal hygiene such as handwashing and drinking clean water [6], as well as by food hygiene [7]. Diarrhoea can be successfully managed and prevented in highly cost-effective ways [8] but in low-resource settings care often remains financially or physically inaccessible. There is a need for more improving accessibility of existing interventions as well as identifying ways to further improve their effectiveness.

The cost of treating and managing diarrhoea can significantly impact providers, patients, and their households as well as the whole society [9–11]. The aim of most cost of illness (COI) studies is to identify and measure costs of a particular disease, which may include direct and indirect costs, as well as intangible costs such as emotional hardship which are hard to monetise. The COI of a diarrhoea case encompasses the direct treatment expenses and non-medical expenses such as travel/food, and the indirect costs such as productivity loss or loss of income [12]. Direct costs can be catastrophic for households with no other option than to pay out-of-pocket, often leading to reduced expenditure on other necessities such as food and education [13]. Indirect and non-medical costs are also an important burden on households, with a study in Malawi finding that nearly half of diarrhoea COI was attributed to these costs [14]. A recent systematic review of the COI for childhood diarrhoea in LMICs found a median cost of US$ 160 per inpatient episode (2015 prices) and US$ 37 per outpatient episode [12]. Factors associated with COI should be explored to help identify interventions to reduce costs [15–17]. COI estimates are important inputs into other studies, e.g. economic burden calculations, catastrophic health expenditure and cost-effectiveness studies [18,19].

In the Democratic Republic of the Congo (DRC), there are estimated to be 45 million diarrhoea episodes of children below 5 years every year [2], resulting in 19,000 deaths [2]. Despite this, we are not aware of any studies of the COI of diarrhoea in DRC. COI can vary between countries and settings for many reasons such as macroeconomic factors, care-seeking patterns, and health system organisation. Therefore, country- or setting-specific estimates are often more accurate than extrapolating estimates from other [12]. There have been COI studies in DRC for other high-burden diseases affecting children such as malaria [20,21]. A diarrhoea COI study in DRC would provide information that is useful locally, as well as support other studies in DRC, but it would also add to the global literature. Our study aims to estimate the economic cost per case of diarrhoea amongst individuals of any age in Uvira, DRC, from a societal perspective.

## Methods

### Ethics statement

Ethical approval to conduct this was obtained from the School of Public Health of the University of Kinshasa (ref. ESP/CE/143R/2021) and the Research Ethics Committee at the London School of Hygiene & Tropical Medicine (ref. 8913). All methods were performed in accordance with relevant local and interntional guidelines and regulations (e.g Declaration of Helsinki). Informed consent was documented in writing for each participant prior to the administration of the questionnaire.

### Setting

This study was carried out in the city of Uvira in the Democratic Republic of the Congo.

Uvira’s population was around 280,000 in 2020 according to official census data, and cholera is endemic with regular seasonal outbreaks [22]. The DRC health system has a decentralized three-tier structure with low levels of public funding, hence the health system relies heavily on user fees. The rationale for working in Uvira was our our study was undertaken as part of a broader study of domestic water-related practices, within the framework of a stepped-wedge cluster randomised trial (SW-CRT) taking place in the city [23]. Treatment of severe acute diarrhoea is available for free at the Cholera Treatment Centre (CTC) within the Uvira general hospital and the Cholera Treatment Unit at Kalundu Health center. Treatment for diarrhoea may also be sought at private health centres or pharmacies. Since 2013, the water utility REGIDESO has invested approximately 15 million euros to improve water supply infrastructure, through support from the French Development Agency (AFD), the European Union, and the Veolia foundation. The works carried out until 2022 include modernisation of the treatment station, new reservoir construction, and installation or rehabilitation of domestic water connections for 2,368 households Oral cholera vaccine was distributed through mass vaccination campaigns in 2020 (and does not form part of diarrhoea treatment).

### Study design and participants

Our study was cross-sectional and applied a retrospective approach to calculate the COI per episode of diarrhoea, based on a household survey representative of the city. Economic costs were estimated from the societal perspective, meaning costs are included no matter who bears them. Eligible study participants were adults aged 18 or over able to answer questions about water-related practices, but participants were asked about 7-day diarrhoea prevalence for all household members.

### Data collection and sample size

Our sample size was determined by the broader study, which had a target enrolment of 500 households [23]. Households were sampled according to simple random spatial sampling, based on the extraction of buildings in Uvira from a high-resolution satellite image. We used ArcGIS (version 10.8.1) to select 800 points randomly distributed across 12 out of 16 clusters in the SW-CRT. These clusters encompass approximately 90% of Uvira’s population, so our sample is broadly representative of the city. Upon arriving in the area, fieldworkers identified geo-locations using OSMAND software on a tablet. Fieldworkers collected data in September 2021 using ODK Collect software. In the absence of the inhabitants, the interviewers visited each residential building up to three times to enrol and administer the questionnaire. The primary respondent was the person aged 18 years or above best-informed about water practices in the household, if available, else any household memberaged 18 years or above.

The final sample was 528 households, within which 2,820 individuals resided and reported diarrhoea prevalence information. Of those individuals, 175 people from 123 different households were reported as having had diarrhoea during the previous seven days (prevalence 6.2%). The effective precision with n=528 households allows estimating the prevalence of an indicator reported by 30% of the population with an accuracy of +/- 4% at 95% confidence level. Since diarrhoea was reported by patients or family members in our dataset, the diarrheogenic pathogen is not known. However a recent study of n=292 patients at the Uvira CTC used polymerase chain reaction to detect toxigenic Vibrio cholerae in 38% of CTC patients [24]. Other common pathogens were enterotoxigenic Escherichia coli (36%) and Cryptosporidium (28%).

### Cost evaluation

Costing components included in this study were user fees for medicine and services (direct medical cost), transport (direct non-medical cost) and productivity losses (indirect cost).We did not aim to include intangible costs because of limited questionnaire space. Direct costs incurred by households were determined by the total expenses reported by study participants (patients and/or caregivers, depending on the respondent). Methods for provider direct costs depended on the provider. For treatment provided by the CTC, which is free, we estimated the economic cost of treating the average case via documents provided by CTC staff and informed by discussions with them. These documents included staff salary records, requisitions for materials, and the numbers of diarrhoea cases treated per month (Table A in S1 Text). We also applied this estimated cost to those who sought care at Kalundu Health Center, which is also free. For treatment by other providers, which is not free, the out-of-pocket expenditure reported by households for medicines and fees was assumed to approximate the economic cost of provision. People who spent 1 or more nights in a healthcare facility (HCF) were classified as inpatients. Non-inpatients were classified as “others” rather than outpatients to avoid confusion between those who received pharmacy treatment as opposed to outpatient HCF treatment.

For indirect costs, the human capital approach was used to value time lost by patients and caregivers. The value of lost time was estimated by multiplying the number of days lost due to diarrhoea illness by a price for time. If the patient or caregiver normally earned a wage, their time was valued at 100% of the wage they reported. If they were unwaged, their time was valued at 50% of the median wage in the sample (25% if aged 5-16) [25]. If they were aged under-5, their time was valued at zero. Given the uncertainty around opportunity cost of time, we include a sensitivity analysis where wages are worth half of these values.

The total cost was computed as the sum of direct and indirect costs over the duration of the case. We report differences in mean costs between age groups, interpreting p<0.05 as evidence of statistical significance. Costs were collected in Congolese Francs (CDF) or United States Dollars (US$) depending on the preference of respondents per cost item, since US$ are widely used in the setting. For analysis, costs were converted to 2021 CDF (the year of the study, so no inflation adjustment required) with headline results also reported in US$ at the exchange rate available in Uvira at the time of the survey (1 US$ = 1989 CF). During data cleaning we aimed to identify and clean outliers that were obviously coding errors, but we did not adjust for outliers because they are costs that people have reported and cost data are often skewed.

### Statistical analysis

We used descriptive statistics to summarise all socio-demographic, economic and clinical variables. We disaggregate demographic data and other results by “under 5 years” and “five years and above” because many studies of diarrhoea which might use our results focus on under-5 children only.

We used principal component analysis (PCA) of asset ownership variables to generate a wealth index and quintiles for the whole household sample [26] (Supplementary Material B). We generated a concentration index (CIN) based on the wealth index, using Stata’s *conindex* command to measure socio-economic inequalities in cost among the diarrhoea patients. The CIN ranges between -1 and 1, and captures the extent to which costs differ across diarrhoea participants ranked by asset index scores [27]. We estimated a mean monthly household income of approximately US$ 88 (CDF 175,500) based on a household consumption survey in nearby Bukavu in May 2021 [28] and use this to explore COI as a percentage of income.

We estimated scores for the Household Water Insecurity Experiences (HWISE) scale [29,30]. HWISE comprises 12 questions, validated in many countries including DRC, resulting in a total score ranging 0-36. Higher scores indicate greater water insecurity, with a cut off of ≥12 indicating a state of water insecurity. All statistical analysis was conducted in Stata version 17 and we considered p-values <0.05 as providing statistically significant evidence of association.

### Regression to explore factors associated with higher costs

To assess factors associated with higher costs, we performed a regression analysis. Since the cost data were highly skewed, we used a generalised linear model with gamma family and log link. Gamma distributions are commonly used to analyse skewed cost data. Compared to other specifications considered, this approach had the lowest Bayesian Information Criterion and had normally distributed residuals. Due to missing data for some covariates, we applied multiple imputation by chained equations with 20 imputations [31]. We clustered standard errors at the household level to account for multiple cases in some households.

In identifying covariates to include, we were guided by the literature on variables associated with the cost of diarrhoeal illness [12,16,32–35], namely the case’s sex, age category and wealth quintile. We also included covariates to explore the role of oral cholera vaccine (OCV) status and of water supply, e.g. the type of water service used for drinking (piped on-premises, piped to public or neighbour’s tap, or unimproved), and water insecurity (binary HWISE status). We excluded variables like inpatient status, provider facility type, and caregiver days because they are part of the cost function itself.

## Results

A total of 175 individuals, from 128 different households, were reported to be suffering from diarrhoea in the past 7 days and were included in the study (**Table 1**). About half of participants were women (56%). The mean age of participants was 21 years, with 81% aged five years or above. About half (49%) of participants came from the poorest two quintiles in the city. The vast majority sought care (91%) and, amongst those, 64% visited a pharmacy and 21% a public or private healthcare facility (HCF). Only 18% of participants spent one or more nights in a HCF (inpatients). About half (43%) had received at least one dose of oral cholera vaccine. About 12% used on-premises piped water and 31% off-premises piped water, with almost all the remainder drinking water from the river or lake. A majority (64%) lived in water insecure households according to HWISE.

**Table 1:** Socio-demographic and clinical characteristics of people with diarrhoea in the past 7 days.

Demographic	All; n(%)	Under 5 years; n (%)	Five years and above; n (%)
Sex;			
Female	98 (56)	16 (50)	78 (57)
Male	76 (44)	16 (50)	58 (43)
Age (years)			
Mean	20.6	2.1	25.2
(SD, Median)	(18.5, 15)	(1.0, 2)	(17.9, 20)
Socio-economic status			
Poorest	41 (23)	9 (27)	29 (21)
Poorer	45 (26)	9 (27)	35 (26)
Middle	32 (18)	9 (27)	23 (17)
Richer	31 (18)	1 (4)	28 (21)
Richest	26 (15)	5 (15)	21 (15)
Provider*			
Public HCF	20 (11)	3 (9)	17 (12)
Private HCF	16 (9)	1 (3)	15 (11)
Pharmacy	112 (64)	24 (73)	83 (61)
Traditional healer	10 (6)	1 (3)	9 (7)
Others	1 (1)	0 (0)	1 (1)
No care sought	16 (9)	4 (12)	11 (8)
Oral cholera vaccine			
At least one dose Received	75 (43)	10 (32)	63 (47)
Not received	96 (55)	21 (68)	71 (53)
Type of patient			
Inpatient	31 (18)	4 (12)	26 (19)
Other	144 (82)	29 (88)	110 (81)
Patient lost days**			
Mean (n=142)	3.3	3.3	2.9
(SD, Median)	(2.2, 2)	(2.2,2)	(1.8,1)
Caregiver lost days**			
Mean (n=55)	4.1	3.9	4.2
(SD, Median)	(2.3, 5)	(2.1, 3)	(2.4, 5)
Caregiver lost wages			
wage loss	55 (31)	10 (31)	43 (46)
No wage loss	120 (69)	22 (69)	51 (54)
Water Security			
water insecure	88 (64)	20 (71)	67 (63)
water secure	50 (36)	8 (29)	40 (38)
Drinking water source			
Improved on premises	21 (12)	5 (15)	15 (11)
Improved off premises	52 (30)	8 (24)	44 (32)
Unimproved	102 (58)	20 (61)	77 (57)

Data shown are frequency (%) unless otherwise stated. 6 participants are missing age data, so “All” column is not sum of other columns

NB: if a total n per variable does not sum to 175 then the remainder is due to missing data

*only one provider could be selected, which was the main place from which care was sought.

**refers to patients’ lost time being sick and caregivers’ lost time looking after patients. Means including zeroes (those with no lost time) are reported in Table B in S1 Text.

The mean economic COI was CDF 33,816 per diarrhoea episode (US$ 17.0) (**Table 2**). The median was 14,000 (US$ 7.0) with inter-quartile range 6,484 (US$ 3.3) to 40,694 (US$ 20.5). The mean cost for people aged five years and above was CDF 39,177 per episode (US$ 19.7) while for those under five was lower at CDF 12,875 per episode (US$ 6.5)(p=0.020). The average duration of illness was 4 days, meaning patients (and caregivers, if applicable) lose 4 productive days per episode (sensitivity analysis on wage in Table C in S1 Text). Mean out-of-pocket financial cost was CDF 15,579 (US$ 7.8), or about 9% of the estimated average monthly income of households (US$ 88), and the median was CDF 3,000 (US$ 1.5). The average indirect cost represents about 54% of the total cost. Mean total costs were considerably higher than medians, due to outliers with much higher indirect costs. The mean cost for inpatients was CDF 78,573 (US$ 39.5), median CDF 62,890 (US$ 31.6), while for others it was CDF 24,181 (US$ 12.2), median CDF 10,542 (US$ 5.3). Average provider cost for CTC per patient was CDF 71,910 (US$ 36.2).

**Table 2:** Average cost by age and cost components in 2021 Congolese Francs (CDF).

	Under five years (n=33)		Five years and above (n=136)		Overall (n=175)	
	Mean (SD)	Median (IQR)	Mean (SD)	Median (IQR)	Mean (SD)	Median (IQR)
Direct Medical Cost (fees/medicines)	12,948	3,000	15,621	4,000	14,708	3,500
	(24,787)	(8,945)	(28,045)	(18,890)	(26,983)	(10,000)
Direct Non-Medical Cost (travel)	1,022	0	2,635	0	2,243	0
	(2,372)	(500)	(11,697)	(1,000)	(10,391)	(1,000)
Indirect Cost (productivity & wage loss)	0	0	22,442	10,968	18,237	8,226
	(0)	(0)	(50,272)	(16,060)	(45,474)	(16,452)
Total economic cost	12,875	2,500	39,177	19,073	33,816	14,000
	(24,483)	(8,945)	(62,868)	(34,697)	(57,654)	(34,210)
Out-of-pocket financial cost	12,875	2,500	16,735	3,500	15,579	3,000
	(24,483)	(8,945)	(32,423)	(16,445)	(30,555)	(10,000)

SD standard deviation

IQR interquartile range (difference between 25^th^ and 75^th^ percentiles)

The concentration index is positive (0.120, p=0.099), indicating that cost is more concentrated among wealthier individuals with higher asset scores (Fig A in S1 Text). This finding is supported by the regression analysis (Table 3), with households from the richest quintile having higher COI than the poorest (p=0.048). The regression analysis also revealed that individuals living in water insecure households had higher COI than those in water secure households, and individuals aged over five years had higher COI than under-fives (**Table 3**). For categorical variables, the value of the exponentiated coefficient minus 1 is interpreted as an approximate percentage change. For example, people aged 15-49 experienced a COI which was approximately 274% higher than under-5s (e^1.318^ – 1 = 2.74).

**Table 3:** Generalised linear regression model with gamma family and log link.

Total cost	Coefficient	[95% conf. interval]	P-Value
Age category (ref. 0-5)			
5-14	0.787	[0.141-1.443]	0.017*
15-49	1.318	[0.716-1.919]	<0.001*
50+	1.107	[0.367-1.828]	0.003*
Gender			
Male (ref. female)	-0.255	[-0.644-0.133]	0.198
Water sources (ref. improved on premises)			
Improved off premises	0.571	[-0.133-1.273]	0.112
Unimproved	0.584	[-0.002-1.171]	0.051
Socioeconomic status (ref. Poorest)			
Poorer	0.348	[-0.281-0.977]	0.278
Middle	-0.227	[-0.832-0.379]	0.463
Richer	0.155	[-0.532-0.843]	0.659
Richest	0.670	[0.058-1.334]	0.048
Water Security			
Insecure (ref. secure)	0.637	[0.053-1.221]	0.032*
Sanitation			
Improved (ref. unimproved)	-0.160	[-0.570-0.249]	0.442

Dependent variable = Total economic cost of illness

Ref. = reference category compared to

* indicates statistical significance (p<0.05)

## Discussion

In this cost of illness (COI) study applying a cross-sectional survey-based approach approximately representative of the city of Uvira, we find that the mean economic COI for an episode of diarrhoea was 33,816 Congolese Francs (CDF) (US$ 17.0). The mean out-of-pocket financial cost (CDF 15,579, US$ 7.8) represents 9% of the average monthly income of households, making it a substantial burden relative to their income. We are not aware of any previous studies on the cost of illness of diarrhoea in DRC, meaning our results can be a useful input into economic evaluations and economic burden estimates in the country as well as contribute to policy and investment strategies in similar cities.

Of the under-5 children with diarrhoea in our sample, only 12% sought advice or treatment from a health provider (excluding pharmacies, shops, and traditional healers). This was slightly lower than the 2018 Multiple Indicator Cluster Survey (MICS) estimate for South Kivu which reports 20% of under-5s with diarrhoea seeking care from (public or private) health centre or health service provider excluding pharmacies [36]. The low healthcare seeking behaviour in our study might be due to the following reasons: i) the relatively high density of pharmacies may partly explain this finding, ii) Chronic insecurity stemming from protracted conflict in and near Uvira often makes people reluctant to stray far from home, especially at night. It is also possible that, only one year into the Covid-19 pandemic at the time of the survey, people remained scared to go to hospitals [37], and iii) With limited access to healthcare services, people resort to informal facilities, self-medication and pharmacies. Hence, the role and widespread use of informal care structures should be carefully considered in designing public health policies and interventions to reduce the burden of diarrhoeal disease. Direct medical cost accounts for 43% of the total economic cost on average (98% of the out-of-pocket financial cost), and the largest contributor to direct costs was drugs and medical supplies (86%) as opposed to transport. This could be explained by the majority of the people with diarrhoea (64%) seeking care at pharmacies. For a mild case, visiting a pharmacy is quick and relatively cheap compared to visiting a health centre, where a doctor may refer you back to a pharmacy anyway. It is likely that the average case seeking care at a HCF is more severe than the average case visiting a pharmacy.

Our estimate likely represents a lower bound on the COI of diarrhoea in this setting, for several reasons. First, due to constraints on questionnaire space in a survey with broader objectives, we were only able to include travel cost in our non-medical cost, contrary to other studies which include components such as accommodation, food, etc., making our results an underestimate [15,38]. Second, for treatment by providers other than the CTC, we relied on respondents recall of expenditure on medicines and fees, which may be subject to recall bias in either direction. Third, the CTC records we used (Table A in S1 Text) are relatively comprehensive, covering 18 different consumable items and 23 staff members from guards to doctors. However, some costs of inpatient care associated with overnight stays are not included, e.g. amortised costs of capital equipment and recurrent costs such as utilities

We found lower direct costs for under-5s compared to those above five years, which may be because only 12% of under-5 participants were inpatients compared to 19% of over-5s. In addition, families may spend more to treat members who stand to lose wages when sick [39]. Also in some health facilities tariffs can vary by age, e.g. with paediatric consultations cheaper than for adults. Amongst people with diarrhoea, as opposed to the sample as a whole, a higher proportion of above-fives had been vaccinated against cholera (47%) than those under- five years (32%). Though cholera and non-cholera diarrhoea in Uvira are seasonal, at the time of our survey in September 2021 CTC cholera admissions had been at very low levels (approximately 10 per month) for almost a year. By contrast, in December 2021 there were almost 250 admissions at the CTC. Average COI for diarrhoea may also be higher at times of cholera outbreak, if the average case is more severe than those included in our sample [40].

Comparing our study findings with similar studies in sub–Saharan Africa [12] our mean of US$ 17.0 falls within the range of previous studies. From the societal perspective, the average cost per episode was estimated to be US$ 11.3, US$ 10.6, and US$ 20.8 in Mali, Gambia, and Kenya respectively, after updating to 2021 prices [41]. However, in Bangladesh and Vietnam the cost per episode from societal perspective was US$ 176.2 and US$ 229.7 respectively [16,42]. Direct comparison of these studies should be done with caution considering variation in design, methods, resource utilization, and socio-economic context.

In our study, average indirect cost was about 54% of the total cost. The mean duration of illness in our study was 4 days, in line with the mean (4.3) for community-based cases in a systematic review of diarrhea duration in low- and middle-income countries [43]. Our finding of indirect costs being higher than direct costs on average are contrary to results from the systematic review of diarrhoea COI studies [12]. We envisage several possible explanations. First, the majority of our participants sought care at pharmacies (64%) rather than public or private HCFs (21%), and we sampled participants from the general population rather than only from those presenting at HCFs as some COI studies have done [44–46]. Second, for costs at HCFs other than the CTC, we relied on respondents recall of expenditure on medicines and fees. If such expenditure does not reflect the full direct medical cost, then it will be an underestimate. Most of our participants (13/20) which sought care at a public HCF did not seek care at the CTC. Care at other public facilities is not free but reported expenditure may underestimate costs if care is directly or indirectly subsidised. Third, to value productivity losses we applied the human capital method based on reported wages, while other studies use premature death [11] or friction cost [47]. Diarrhoeal productivity losses are substantial, affecting not only individuals and their households but also businesses. Estimating COI including productivity losses can strengthen the rationale for interventions that reduce the burden of diarrhoeal diseases.

We found slightly lower COI among individuals within lower socio-economic groups, in agreement with some previous diarrhoea COI studies [48] but not others [16]. Such findings on COI and socio-economic status have not always been consistent in COI studies for diseases other than diarrhoea. On the one hand, richer households are likely to attend HCFs that are more expensive, while poor households may postpone care-seeking as a way of coping with illness. On the other hand, poorer households’ diarrhoea episodes may last longer if they avoid treatment or pursue less effective treatment and poorer people are more likely to be undernourished. COI is also likely to be a higher proportion of income for poorer people. The fact that we followed guidance [25] in valuing lost patient/caregiver time according to their reported wage (or 50% of the median wage if unwaged) may have contributed to this finding, as wealthier households reported higher wages and are more likely to be waged. Our study also provides insights into factors associated with higher COI. We found that age category and water security were the variables associated with diarrhoea cost of illness. Age is related to productivity loss, as productive individuals are likely to spend time caring for the patients. Since we adjusted for wealth (which is associated with water service type and water security), our results show that improving water security may reduce the costs associated with the burden of diarrhoeal diseases.

This study has some limitations. First, the survey was primarily designed for objectives other than COI, so we were able to add only 14 COI-related questions. For example, it would have been better to have data on the cost of food/accommodation and we would ideally have asked disaggregated questions on different kinds of medical expenditure (e.g. medicines, consultation fees). This may have prompted the respondent to recall additional expenditure. Second, due to basing the majority of variables on respondent report, the results may be subject to recall bias or other types of bias (e.g. social desirability). Third, with the exception of the CTC, our price estimates were limited to patient/caregiver report of expenditure rather than engaging with HCFs to assess costs on the provider side. As a consequence, these costs may be imprecisely estimated, and we were not able to disaggregate out-of-pocket costs for patients from costs to health facilities. A related limitation is that, in the absence of data, we assumed that costs in the Kalundu health centre were the same as the CTC (though only 2/175 cases attended Kalundu). Also due to relatively low numbers of participants visiting HCFs, it was not possible to evaluate the difference in direct medical cost between private and public facilities as other studies have done [42,49]. Finally, since this was a COI study not an economic burden study, we do not estimate the lost utility of health (e.g. value of DALYs lost to diarrhoea).

Since our study took place at a time when cholera and non-cholera diarrhoea had been at low levels for almost a year and was based on a sample that was representative of Uvira. We also had only seven cases (4%) who had been admitted to CTC. Therefore, results may to some extent be generalizable to similar cities in DRC with a similar distribution of care-seeking. Qualitative studies about diarrhoea care-seeking in urban DRC may help complement our findings [50].

## Conclusion

In this population, the costs of illness arising from diarrhoea remain an important burden on households. The mean out-of-pocket cost per case represents 9% of the average monthly income of the whole household. A 7-day diarrhoea prevalence of 6.2% equates to multiple annual episodes in a given household. Provider-borne costs also represent a burden on the health system. For diarrhoeal diseases such as cholera, a strong emphasis is put on the multisectoral approach, i.e. WASH combined with healthcare, vaccination, community engagement. Similar approaches are relevant for other diarrhoeal pathogens (e.g. vaccination for rotavirus). Multiple interventions working synergistically may reduce the disease burden and associated costs.

The fact that a large share of the cost is due to indirect costs also points to the importance of the duration of disease, and access to adequate treatment can be key to reducing duration. Future research should investigate the cost of diarrhoea illness in rural areas of DRC, and investigate whether pharmacies in DRC cities are providing appropriate treatment for diarrhoea given their widespread use, in the context of rising antimicrobial resistance.

## Supporting information

S1 Text **Table A** contains detailed information on how the provider cost per patient was measured and valued. **Table B** shows a summary of statistics of the comparison between caregiver and patient time loss. **Table C** illustrate the sensitivity analysis of the opportunity cost of time. Finally, **Fig A** shows the distribution of costs across by asset index score.
(DOCX)
