# Supplementary material for: Economic cost of a case of diarrhoea in Uvira, Democratic Republic of the Congo: A cost of illness study
Source: PLoS Negl Trop Dis. 2024 Oct 28;18(10):e0011934. doi: 10.1371/journal.pntd.0011934 (PMC11542808; doi:10.1371/journal.pntd.0011934)
Supplement: S1 Text — Table A contains detailed information on how the provider cost per patient was measured and valued. Table B shows a summary of statistics of the comparison between caregiver and patient time loss. Table C illustrate the sensitivity analysis of the opportunity cost of time. Finally, Fig A shows the distribution of costs across by asset index score. (DOCX) [file pntd.0011934.s001.docx]

## Cholera Treatment Centre costing

Treatment at the CTC is free, so we estimated the economic cost of treating the average case of diarrhoea via documents described below provided by CTC staff. Estimation was also refined through informal discussions with CTC staff. For the total cost of illness, this was summed with direct non-medical costs (transport, food) and indirect costs (caregiver/patient time).

The CTC only treats severe acute diarrhoea (suspected cholera). To calculate staff costs, we obtained the list of all staff contributing to the service at the CTC, namely doctors, nurses, hygienists, cleaners and other casual workers, during 1^st^ June – 31^st^ August 2019. Many nurses (and doctors) in DRC have two sources of monthly income: (i) salaries from the hospital; (ii) monthly incentive known here as “prime de risque” from the central government. For many doctors for instance, the “prime de risque” is higher than the salary. Currently, junior doctors have a “prime de risque” of about USD 700 per month (1,400,000 Congolese Francs), far higher than local salaries in many hospitals. We summed their monthly salary with any top-up payments based on what was available at disposal. To calculate consumable costs, we obtained the quarterly requisition for resources including medicines (e.g., doxycycline, oral rehydration solution), equipment (e.g. catheters) and personal protective equipment (e.g. gloves). The requisition provides data on quantities, and market prices were provided by a pharmacist. We multiplied these together and then divided by three to arrive at a monthly value. To estimate the cost per patient, we obtained data on weekly inpatient numbers, and calculated the average number of monthly cases during 2018-2020. We then estimated direct medical cost per patient by dividing the total month cost by the average number of monthly patients (88.9).

**Table A:** Cholera Treatment Centre costing

Item Cost (US$ 2019)

| Monthly staff costs | 2,115 |
| --- | --- |
| Monthly consumable costs | 1,106 |
| Monthly total cost | 3,221 |
|  |  |
| Cost per patient | 36.2 |

## Principal Component Analysis (PCA)

Based on the items included in the questionnaire, commonly used items and assets were considered in the analysis. PCA works best when variable are correlated and varies across households. We recoded categorical variables into binary variables to make them suitable for PCA [1]. The full list of items is as follows: house wall and floor materials, cooking fuel, internet access, and ownership of assets (wall, floor, electricity, coal ,wood ,leave sticks ,dung , oil, gas, radio, cell phone television, fridge, computer, satellite dish, tablet, internet access, bicycle, motorbike, car, and moto taxi) [1]. We started by creating global macros for all asset items, describing, summarizing, and assessing correlation. Then, we plotted the eigenvalues, followed by component rotations and finally scores of the components using the prediction command in Stata. We measured the sampling adequacy value using the Kaiser-Meyer-Olkin (KMO), and our sample was above the threshold value for satisfaction..

**Fig A:** Lorenz curve

The below Lorenz curve shows the distribution of costs across by asset index score. The concentration index is positive hence the curve is >0 showing majority with high assets score incur most diarrhoea costs.

Figure 1 cost of diarrhea illness by assets score

## Patients’ and Caregiver lost time

**Table B :** Detailed summary of patients and caregiver time loss.

|  | Observation | Mean | Standard deviation |  |  |
| --- | --- | --- | --- | --- | --- |
| Patients | 142 | 3.26 | 2.247 |  |  |
| Caregivers | 55 | 4.07 | 2.35 |  |  |
| Patients | | | Caregivers (amongst those experiencing loss) | | |
| Days | Frequency | % | Days | Frequency | % |
| 0 | 6 | 4.23 | 0 | - | - |
| 1 | 21 | 14.79 | 1 | 8 | 14.55 |
| 2 | 38 | 26.79 | 2 | 11 | 20.00 |
| 3 | 30 | 21.13 | 3 | 8 | 14.55 |
| 4 | 13 | 9.15 | 4 | 4 | 7.27 |
| 5 | 9 | 6.34 | 5 | 8 | 14.55 |
| 6 | 6 | 4.23 | 6 | 1 | 1.82 |
| 7 | 17 | 11.97 | 7 | 14 | 25.45 |
| 10 | 1 | 0.70 | 10 | 1 | 1.82 |
| 14 | 1 | 0.70 |  |  |  |

**Table C:** Sensitivity analysis

A sensitivity analysis where the opportunity cost of time is actually worth half the assumed value we attribute to it in the base case (e.g. time of waged people valued at 50% of their wage. Unwaged people’s time valued at 25% of the median wage in the sample (12.5% if aged 5-16)

|  | **Waged adults**  **CDF** | **Unwaged adults(50%)**  **CDF** | **Aged (5-16yrs) 25%**  **CDF** | **Total** |
| --- | --- | --- | --- | --- |
| **Indirect costs** |  |  |  |  |
| Mean | 22,428 | 15,849 | 7,245 | 18,060 |
| SD | (58,509) | (47,543) | (10,476) | (45,940) |
| Median | 17,113 | 10,968 | 8,226 | 8,226 |
| IQR | (18,645) | (12,661) | (6,855) | (16,452) |

**References**

1. Vyas S, Kumaranayake L. Constructing socio-economic status indices: how to use principal components analysis. Health Policy and Planning. 2006;21(6):459-68. doi: 10.1093/heapol/czl029.
